# Sensitivity Improvement of Highly Stretchable Capacitive Strain Sensors by Hierarchical Auxetic Structures

**DOI:** 10.3389/frobt.2019.00127

**Published:** 2019-11-22

**Authors:** Jun Shintake, Toshiaki Nagai, Keita Ogishima

**Affiliations:** Department of Mechanical and Intelligent Systems Engineering, School of Informatics and Engineering, University of Electro-Communications, Chofu, Japan

**Keywords:** stretchable, capacitive, strain sensors, auxetic structures, soft robotics, wearable devices

## Abstract

Highly stretchable sensors that can detect large strains are useful in deformable systems, such as soft robots and wearable devices. For stretchable strain sensors, two types of sensing methods exist, namely, resistive and capacitive. Capacitive sensing has several advantages over the resistive type, such as high linearity, repeatability, and low hysteresis. However, the sensitivity (gauge factor) of capacitive strain sensors is theoretically limited to 1, which is much lower than that of the resistive-type sensors. The objective of this study is to improve the sensitivity of highly stretchable capacitive strain sensors by integrating hierarchical auxetic structures into them. Auxetic structures have a negative Poisson's ratio that causes increase in change in capacitance with applied strains, and thereby improving sensitivity. In order to prove this concept, we fabricate and characterize two sensor samples with planar dimensions 60 mm × 16 mm. The samples have an acrylic elastomer (3M, VHB 4905) as the dielectric layer and a liquid metal (eutectic gallium-indium) for electrodes. On both sides of the sensor samples, hierarchical auxetic structures made of a silicone elastomer (Dow Corning, Sylgard 184) are attached. The samples are tested under strains up to 50% and the experimental results show that the sensitivity of the sensor with the auxetic structure exceeds the theoretical limit. In addition, it is observed that the sensitivity of this sensor is roughly two times higher than that of a sensor without the auxetic structure, while maintaining high linearity (*R*^2^ = 0.995), repeatability (≥10^4^ cycles), and low hysteresis.

## Introduction

Highly stretchable strain sensors, which detect large strains, are an important element for sensing deformations of compliant systems, to assess their status and performance control. These soft matter-based systems include robots and wearable devices (Polygerinos et al., 2017; Rich et al., 2018; Shintake et al., 2018a), with applications in exploration (Katzschmann et al., 2018), manipulation (Shintake et al., 2016), virtual reality (Suzuki et al., 2016), and human monitoring (Bartlett et al., 2016) to name a few. Researchers have developed highly stretchable strain sensors using various materials and fabrication methods, as summarized in a review article by Amjadi et al. (2016).

There are mainly two types of sensing modes available for highly stretchable strain sensors: resistive and capacitive modes. Resistive sensing is based on the piezo-resistive effect where strain causes deformation of the electrodes, and subsequent change in their electrical resistivity. Capacitive sensing relies on changes in capacitance between a pair of electrodes, sandwiching a dielectric material. Strain changes the area of the electrodes and the thickness of the dielectric material, resulting in a variation of the capacitance. As discussed in the literature on strain sensors (Amjadi et al., 2016; Shintake et al., 2018b), although the resistive-type strain sensors exhibit high sensitivity (gauge factor, GF), they have large hysteresis and a non-linear response. On the other hand, the capacitive-type strain sensors show small hysteresis and a linear response, thus making them a promising solution for the soft matter-based systems.

However, the sensitivity of capacitive strain sensors is low [theoretically 1 (Amjadi et al., 2016; Shintake et al., 2018b)], which renders them useless for certain applications, for instance, when a high resolution is required in a fixed system specification. So far, only a few studies have reported improvement in sensitivity of the capacitive strain sensors. Nur et al. have developed a sensor with electrodes made from wrinkled gold films and have shown sensitivity of 3.05 with maximum strain of 140% (Nur et al., 2018). Xu et al. have demonstrated that a nano composite membrane consisting of ionic hydrogels and silver nano fibers, exhibits a large change of the capacitance between the electrodes, placed at the edges of the membrane (Xu et al., 2019). In their study, the sensitivity is reported to be 165 with maximum strain of 1,000%. While these studies are successful in improving the sensitivity, they suggest opportunities for further developments. For example, fabrication of wrinkled gold films requires pre-stretch of the substrate and metal deposition, which can be difficult to carry out due to its need for a specialized setup; whereas, hydrogels can suffer from evaporation of aquatic contents that can change their sensing behavior.

In this paper, we describe a method to improve the sensitivity of stretchable capacitive strain sensors. Our approach is to mechanically program the deformation behavior of the sensors by integrating hierarchical auxetic structures into them. Auxetics are a type of meta-material, that exhibit a negative Poisson's ratio (Liu and Hu, 2010; Ren et al., 2018). When integrated into the sensors, auxetic structures increase the amount of change in the capacitance with applied strains, thus leading to a higher sensitivity. For this study, we have fabricated a prototype strain sensor where, the auxetic structure forms an elastomeric layer, and the entire fabrication is done by layer-by-layer process in a room environment. Thus, the structure of our sensor and its fabrication process are relatively simpler, compared to the other studies. The materials used, are elastomers and a liquid metal, which have almost negligible water content, and provide a stable sensor response. Using this prototype, we demonstrate, that the sensitivity of the capacitive strain sensor can be improved by roughly two times, while maintaining the original characteristics, such as high linearity, repeatability, and low hysteresis.

## Sensitivity of Capacitive Strain Sensors

**Figure 1:**
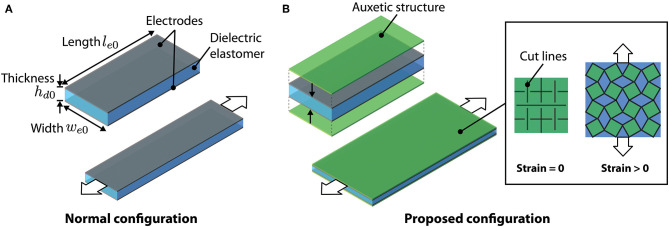
**(A)** Schematics of capacitive-type stretchable strain sensor. **(B)** Schematics of the senor with the hierarchical auxetic structure proposed in this study.

Generally, the structure of highly stretchable capacitive strain sensors is similar to that of a parallel plate capacitor, i.e., it consists of a soft dielectric layer sandwiched between two compliant electrodes, as represented in Figure 1A. With no strain applied, the capacitance of the sensor in the initial state can be expressed as

(1) $$\begin{array}{l} C_{0}=\varepsilon_{0}\varepsilon_{r}\frac{l_{e0}w_{e0}}{h_{d0}}, \end{array}$$

where, ε_0_ is the permittivity of free space, ε_*r*_ the relative permittivity of the dielectric layer, *l*_*e*0_ the initial length of the electrode, *w*_*e*0_ the initial width of the electrode, and *h*_*d*0_ is the initial thickness of the dielectric layer. Upon the application of uniaxial strain, the electrodes of the sensor elongate, their surface area increases and the thickness of the dielectric layer decreases. This leads to changes in the capacitance. Assuming the Poisson's ratio for the dielectric layer and for the electrodes to be the same, the capacitance of the sensor under uniaxial strain can be written as,

(2) $$\begin{array}{l} C=\varepsilon_{0}\varepsilon_{r}\frac{l_{e0}{\left(1+\varepsilon \right)w}_{e0}\left(1-\varepsilon \nu_{2}\right)}{h_{d0}\left(1-\varepsilon \nu_{3}\right)}, \end{array}$$

where, ε is the strain in the loading (length) direction and ν_2_ and ν_3_ are the Poisson's ratio in the width direction and the thickness direction, respectively. Assuming, that the materials used in the sensor are incompressible, like elastomers (i.e., ν_2_ = ν_3_ ≈ 0.5), Equation (2) becomes

(3) $$\begin{array}{l} C=C_{0}\left(1+\varepsilon \right). \end{array}$$

Equation (3) suggests that the response of the sensor is proportional to the applied strain. The sensitivity (gauge factor, GF) of the sensor is obtained as

(4) $$\begin{array}{l} GF=\frac{C-C_{0}}{C_{0}\varepsilon }=1. \end{array}$$

Thus, Equation (4) indicates that the sensitivity of capacitive strain sensors with normal configuration is equal to 1.

## Materials and Methods

As discussed above, when the principal strain is applied, the capacitance of the sensor gets larger due to the increase in electrode area and decrease in the thickness of the dielectric layer, as represented in Figure 1A. The Poisson's ratio of the sensor structure is normally close to 0.5, which reduces the width of the electrode and essentially limits the sensitivity theoretically to 1. In this study, the idea is to integrate auxetic structures into the sensor architecture for improving its sensitivity. In the proposed sensor configuration illustrated in Figure 1B, auxetic structures are attached on both sides of the sensor. Under application of the principal strain, the negative Poisson's ratio of the auxetic structures causes the width to be increased during sensor deformation, unlike the normal sensors (Figure 1A) where the width reduces in a similar situation. This means that ν_2_ in Equation (2) takes a negative value and no longer equals ν_3_. The increased rate of the width change causes increased electrode area expansion and subsequent thickness reduction, therefore affecting the capacitive change, and results in high sensitivity.

As for the geometry of the auxetic structure, we employed the hierarchical design described in the study by Tang et al. (2015). The deformation of this geometry is depicted in Figure 1B inset. When stretched, the segments in the auxetic structure rotate, resulting in an extension perpendicular to the direction of the applied strain. This deformation characteristic leads to an increase in the width of the sensor instead of the normal decrease in width. Apart from the reasons mentioned above, we chose this design as it exhibits a constant negative Poisson's ratio until 70% of linear strain.

**Figure 2:**
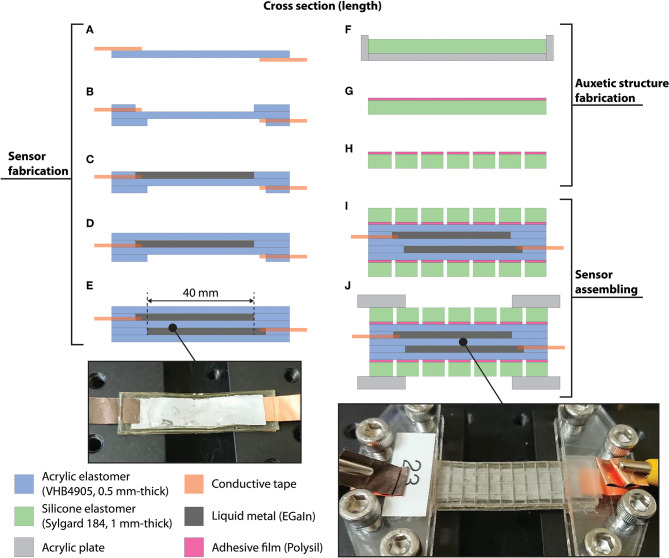
Fabrication process of the sensor sample. **(A–E)** Fabrication of the base sensor. **(A)** A sheet of the acrylic layer is cut and a conductive tape is placed on both sides of the layer. **(B)** Additional acrylic layers with the electrode shape are attached. **(C)** EGaIn is filled and **(D)** sealed with another acrylic layer. **(E)** Repeating the steps **(B–D)** on the other side. **(F–H)** Fabrication of the auxetic structure. **(F)** The silicone elastomer is poured into a mold and cured. **(G)** A silicone adhesive film is attached onto the silicone elastomer. **(H)** The whole sheet is cut. **(I,J)** Assembling of the parts. **(I)** The auxetic structures are attached on both sides of the base sensor. **(J)** Holders are placed.

The focus of this paper is to prove the aforementioned concept through the characterization of a physical sensor sample. The sensor sample is composed of several layers comprising of the dielectric, electrodes, and auxetic structures, respectively. Figure 2 details the fabrication process that is done in three parts: (1) fabrication of the base sensor, (2) fabrication of the auxetic structure, and (3) assembling of these parts. We used a 0.5 mm-thick acrylic elastomer layer (3M, VHB 4905) as the dielectric and the structure of the base sensor, a silicone elastomer (Dow Corning, Sylgard 184) as the auxetic structure, and a liquid metal [eutectic gallium-indium (EGaIn)] (Dickey et al., 2008; Dickey, 2017) for the electrodes. These materials were integrated layer-by-layer. It can be seen in the figure that in the base sensor, the liquid metal is encapsulated by the acrylic elastomer layers.

Figures 2A–E show the fabrication process of the base sensor. (Figure 2A) First, a sheet of the acrylic layer for the dielectric was cut using a laser-engraving machine, and a conductive tape was placed on both sides of the layer. (Figure 2B) Then, additional acrylic layers that created a hollow space corresponding to the electrode shape, were attached. Bonding of the layers was assured by their intrinsic stiction. (Figure 2C) After this, EGaIn was filled manually in the hole and (Figure 2D) sealed with another acrylic layer. (Figure 2E) The steps (Figures 2B–D) were repeated on the other side of the dielectric layer, and the base sensor was complete. The inset picture in Figure 2E shows one of the fabricated sensors.

Figures 2F–H show the fabrication process of the auxetic structure. (Figure 2F) First, the silicone elastomer was mixed with a curing agent in the weight ratio of 10:1, and this mixture was poured into a mold made from an acrylic plate. The mixture was then cured in an oven at 60°C for 2 h, which formed a membrane of thickness 1.0 mm. (Figure 2G) Subsequently, a silicone adhesive film (Taiyo Wire Cloth, Polysil) was attached onto the membrane. (Figure 2H) Finally, the whole sheet was cut using the laser-engraving machine, and the auxetic structure parts were fabricated.

Figures 2I,J show the assembling process. (Figure 2I) The auxetic structures were attached on both sides of the base sensor. (Figure 2J) Holders made of an acrylic plate were placed. The inset picture in Figure 2J shows one such fabricated sensor that is ready to be tested. The effective length of the sensor where the electrodes overlap was 40 mm, with the outline dimensions of 60 mm × 16 mm.

In order to investigate the effect of auxetic structure on the improvement of sensitivity, we performed the characterization on a fabricated prototype. In the characterization, two different sensor samples were prepared. One was a sensor with the auxetic structure, and the other was without the auxetic structure. Each sample was attached to a linear motorized stage (Zaber, X-LRT-1500) and up to 50% of strain was applied with 5% steps for capacitance measurement by an LCR meter (TEXIO, LCR6000). With the same setup, repeatability of the sensor response was characterized by applying cyclic strain of 50% (strain speed 25%/s). Supplementary Video 1 shows the sensor being tested under repeated strain. In the characterization, we put our focus on exploring sensitivity as well as other characteristics such as linearity and hysteresis. The sensitivity of the sensor was calculated using Equation (4).

## Results and Discussions

**Figure 3:**
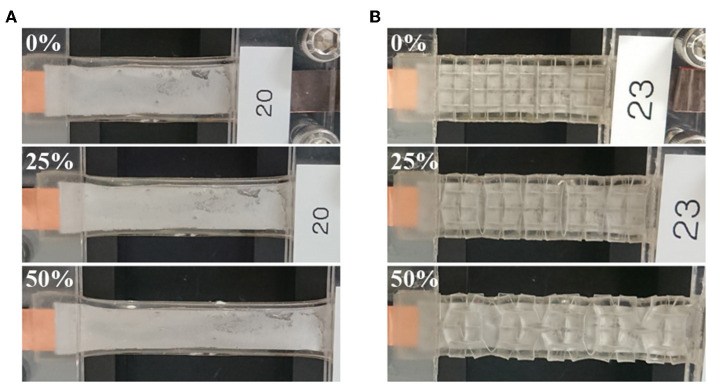
Deformation of the fabricated sensors at the applied strain of 0, 25, and 50% (top view). **(A)** Sensor without the auxetic structure and **(B)** sensor with the auxetic structure.

**Figure 4:**
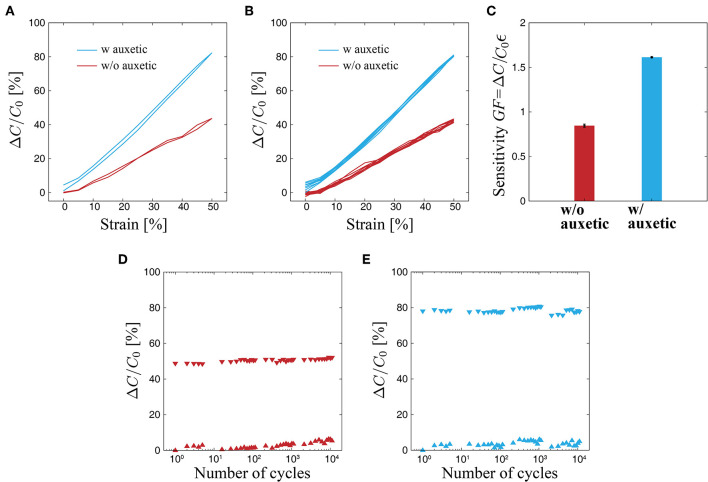
Characterization results of the fabricated sensors. **(A)** Capacitive change as a function of the applied strain for one cycle. **(B)** Capacitive change as a function of the applied strain for five cycles. **(C)** Calculated sensitivity of the sensors. **(D)** Response of the sensor without the auxetic structure for 11,135 cycles. **(E)** Response of the sensor with the auxetic structure for 11,135 cycles.

First, we visually observed the deformation behavior of the sensor samples before starting the characterization. As can be seen in Figures 3A,B, the presence of the auxetic structure makes the electrodes wider with stretching, which is obvious, especially when 50% strain is applied. We then assessed the sensitivity of the sensors. The initial capacitance value of the sensors right after the fabrication was 32.9 pF for the sensor with the auxetic structure and 32.6 pF for the sensor without the auxetic structure, respectively. As plotted in Figure 4A, the sample with the auxetic structure exhibited larger capacitance change compared to the one with normal configuration. The larger change in capacitance indicates a higher sensitivity, which essentially validates the concept of this study.

Moreover, the sensor with the auxetic structure still holds the original characteristics of capacitive sensing, such as high linearity (*R*^2^ = 0.995) and small hysteresis (3.5%). Here, the hysteresis was evaluated as the drift error in the sensor reading at 0% strain, measured before and after the stretch cycles. The two parameters, linearity and hysteresis, are not directly linked to the sensitivity but are important in strain sensors; non-linearity can make the calibration of the device more complex and difficult, whereas large hysteresis results in the irreversible sensing performance (Amjadi et al., 2016). In Equation (2), our sensor should possess a negative value of Poisson's ratio ν_**2**_ in the width direction, due to the auxetic structure property and also ν_**2**_≠ν_**3**_. This changes the form of Equation (3) and predicts the sensor response to be non-linear. The reason why the result shows linearity could be the change of ν_**2**_ with the applied strain. In the proposed configuration, ν_**2**_ is considered a constant regardless of the amount of the strain. However, because the entire deformation of the sensor is determined from the structural interaction between the auxetic structures and other parts, the Poisson's ratio ν_**2**_ may dynamically change with strain, thus resulting in a linear response.

The performance of the sensor was maintained for additional cycles, as shown in Figure 4B. Based on this result, we calculated the sensitivity GF. The obtained value was 1.61 ± 0.01 for the sensor with the auxetic structure, and 0.86 ± 0.02 for that without the auxetic structure, thus indicating that it increased by 1.9 times for the former sensor (Figure 4C). The sensitivity 1.61 obtained in this study is lower than those reported in the other studies: 3.05 (Nur et al., 2018) and 165 (Xu et al., 2019). However, the current design of the sensor is only to validate the concept of this study and it can potentially be optimized for achieving better performance. Moreover, our method also allows implementation of various auxetic geometries available in the literature (Liu and Hu, 2010; Ren et al., 2018), which could further improve sensitivity.

We also characterized the repeatability and robustness of the sensor for a larger number of strain cycles. We applied more than 11,000 strain cycles (50%) to the sensor samples. The result is plotted in Figures 4D,E. As can be seen, both sensors exhibited excellent repeatability and durability, which implies that the presence of the auxetic structure does not influence the repeatability of the sensor response, and maintains high robustness successfully.

To summarize, we have described a method to improve the sensitivity of highly stretchable strain sensors by integrating auxetic structures. The proposed sensor configuration uses the negative Poisson's ratio of auxetic structures that increases the width of the sensor in response to the principal strain in the length direction, unlike the normal sensor configuration where the width reduces. The deformation characteristics of the sensor causes larger capacitive change and improved sensitivity. Through the characterization, we have shown that the fabricated sensors exhibit improved sensitivity, roughly two times of the normal sensors. Aside from higher sensitivity, the sensors also show linearity, low hysteresis, repeatability, and durability. The results validate the feasibility of the use of the auxetic structures for highly stretchable strain sensors and thus contribute to their further development. Lastly, our ongoing work is to further improve the sensitivity by optimizing the geometry of the sensors and to understand their mechanical behavior with the aid of analytical and numerical modeling. As demonstrated in many studies for example in Bartlett et al. (2016); Nur et al. (2018), and Shintake et al. (2018b), strain sensors are able to detect bending deformations when placed on the surface of the target object. Therefore, applications of our sensor will also include soft robotic systems that undergo large bending deformations such as grippers, mobile robots, and wearable devices.

## Data Availability Statement

The raw data supporting the conclusions of this manuscript will be made available by the authors, without undue reservation, to any qualified researcher.

## Author Contributions

JS and KO designed the experiments. KO and TN collected and processed data. Data interpretation was performed by JS and TN. JS wrote the manuscript. All authors have read and approved the final manuscript.

### Conflict of Interest

The authors declare that the research was conducted in the absence of any commercial or financial relationships that could be construed as a potential conflict of interest.

## Supplementary Material

Supplementary Video 1 Deformation of the fabricated sensor.
